# 5-Hydroxymethylcytosine modifications in circulating cell-free DNA: frontiers of cancer detection, monitoring, and prognostic evaluation

**DOI:** 10.1186/s40364-025-00751-9

**Published:** 2025-03-07

**Authors:** Danjun Song, Zhou Zhang, Jiaping Zheng, Wei Zhang, Jiabin Cai

**Affiliations:** 1 Department of Liver Surgery, Zhongshan Hospital (Xiamen Branch), Fudan University, Xiamen, Fujian 361015 China; 2 Department of Liver Surgery and Transplantation, Key Laboratory of Carcinogenesis and Cancer Invasion, Shanghai Key Laboratory of Organ Transplantation, Liver Cancer Institute, Zhongshan Hospital, Fudan University, Zhongshan Hospital, Shanghai, 200032 China; 3 Department of Interventional Therapy, Institute of Hangzhou Medicine, Zhejiang Cancer Hospital, Chinese Academy of Sciences, Hangzhou, Zhejiang 310022 China; 4 Department of Preventive Medicine, Northwestern University Feinberg School of Medicine, Chicago, IL 60611 USA

**Keywords:** 5-hydroxymethylcytosines (5hmC), Cell-free DNA (cfDNA), Liquid biopsy, Clinical application

## Abstract

Developing accurate, clinically convenient, and non-invasive methods for early cancer detection, monitoring, and prognosis assessment is essential for improving patient survival rates, enhancing quality of life, and reducing the socioeconomic burden associated with cancer. This goal is critical in precision oncology. Genetic and epigenetic alterations in circulating cell-free DNA (cfDNA) have emerged as transformative tools for advancing early cancer detection, monitoring, and improving patient outcomes. Among these, 5-hydroxymethylcytosine (5hmC) modifications in circulating cfDNA stand out as promising epigenetic markers, offering insights into cancer initiation, progression, metastasis, and prognosis across various cancer types, such as lung cancer, colorectal cancer, and hepatocellular carcinoma. This review comprehensively explores the biology and sequencing methodologies of 5hmC, emphasizing their potential in cancer screening, diagnosis, treatment monitoring, and prognostic assessment. It highlights recent advancements in cfDNA-derived 5hmC signatures’ applications, addressing their strengths and limitations in the context of clinical translation. Furthermore, this review outlines key challenges and future directions for integrating 5hmC modifications in cfDNA into routine clinical practice, facilitating personalized and non-invasive cancer management.

## Background

Cancer remains one of the leading causes of death worldwide, with incidence and mortality rates projected to rise significantly [1]. By 2050, the global burden of new cancer cases is projected to increase by 77%, reaching approximately 35 million annually compared to 2022 levels [1, 2]. Early detection and screening, especially among asymptomatic and high-risk individuals, are pivotal for improving survival outcomes [3–5]. For example, identifying and treating high-risk precancerous lesions can prevent progression to invasive cancer, while detecting malignancies at earlier stages through secondary prevention strategies is associated with better prognoses and reduced mortality rates.

**Fig. 1:**
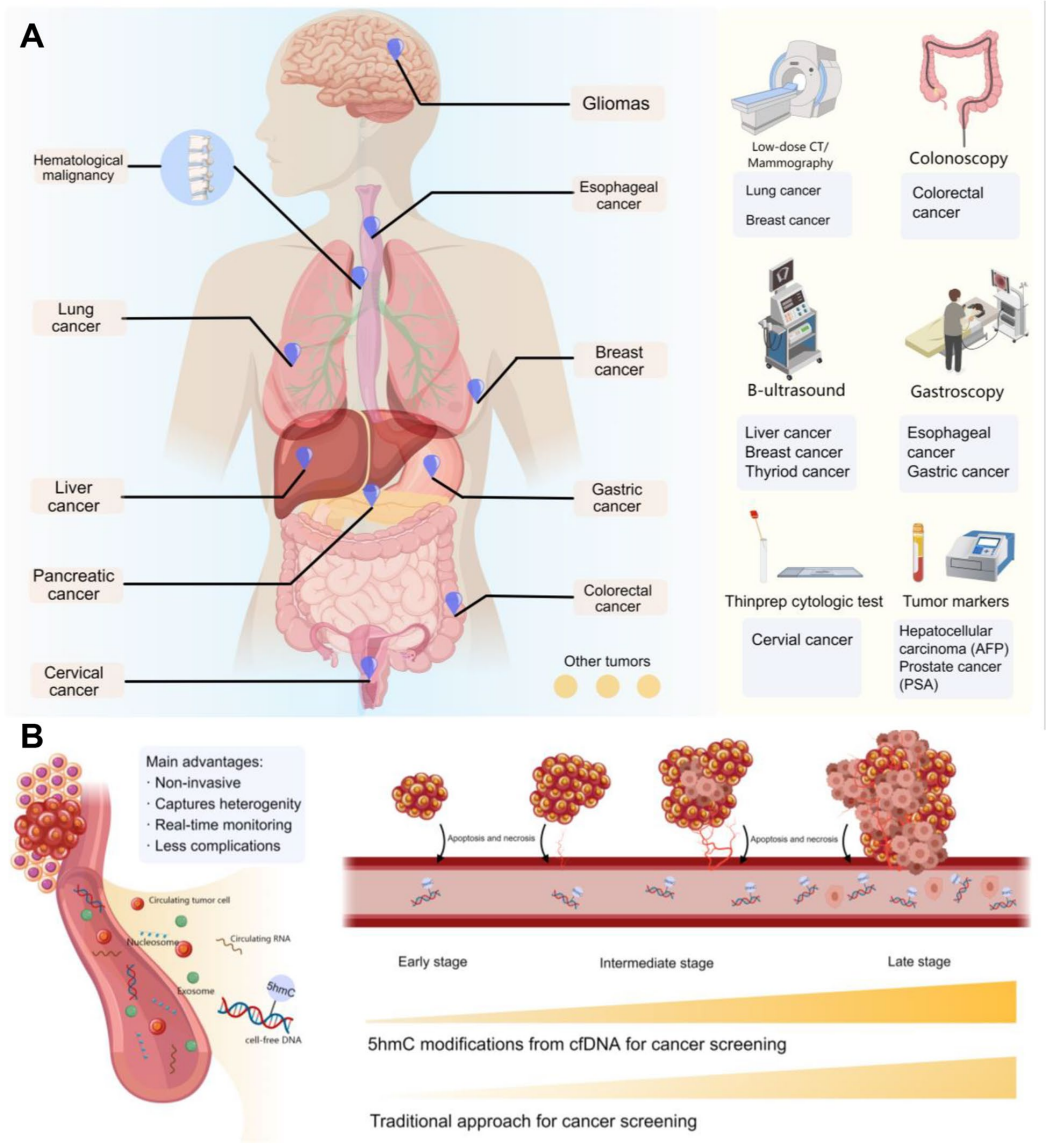
Comparison of 5hmC-based cfDNA liquid biopsy with traditional cancer screening methods. **A** Overview of traditional cancer screening methods, including radiological imaging, endoscopy, and laboratory-based biomarker tests, categorized by cancer types. Traditional screening approaches are often invasive, require specialized equipment, and have variable sensitivity depending on the cancer type. **B** Schematic representation of tumor progression and the role of 5hmC-based cfDNA analysis in cancer detection. The illustration highlights the release of tumor-derived cfDNA into circulation, emphasizing the advantages of 5hmC profiling for non-invasive early detection. The comparison of 5hmC-based detection and traditional approaches illustrates how cfDNA liquid biopsy provides an earlier and less invasive alternative for cancer screening, with improved detection potential across different stages of tumor development. 5hmC, 5-hydroxymethylcytosine; cfDNA, cell-free DNA

Current screening methods, including radiological imaging, endoscopy, and laboratory tests, have significantly enhanced survival rates for cancers, such as lung, gastrointestinal, and breast cancers [6, 7]. However, these modalities face critical limitations: they are often invasive, require complex pre-examination preparations, and exhibit limited sensitivity (Fig. 1) [6]. As a result, patient compliance remains low. Moreover, for certain cancers like neuroendocrine tumors, ovarian cancer, and pancreatic cancer, no effective screening guidelines are established [6]. Tissue biopsy, the gold standard for cancer diagnosis, offers valuable insights for treatment planning but is hindered by its invasiveness, associated complications, and challenges in accessing deep-seated tumors [8, 9]. Consequently, there is a pressing need to develop non-invasive, accurate, and patient-friendly approaches for early cancer detection and diagnosis.

In recent years, epigenetic profiling has gained significant attention for its potential in advancing cancer detection and characterization, as epigenetic alterations play a critical role in cancer onset and progression [10, 11] (Fig. 1). Among these, analyzing specific epigenetic changes in circulating cell-free DNA (cfDNA) has emerged as a powerful non-invasive approach to uncover the underlying molecular mechanisms of cancer and to support early screening, diagnosis, disease monitoring, and prognostic evaluation [10, 12, 13]. In particular, 5-hydroxymethylcytosine (5hmC) modifications have shown great promise as epigenetic biomarkers, closely associated with cancer initiation, progression, metastasis, and prognosis across multiple cancer types [13, 14].

This review explores the potential of 5hmC signatures in circulating cfDNA (cfDNA-5hmC signatures) as a biomarker in multiple cancers for screening, diagnosis, treatment monitoring, and prognostic evaluation. We discuss the biological underpinnings of 5hmC, the technological advances enabling its detection, and clinical utility. Furthermore, we outline the challenges and future directions for advancing the clinical translation of cfDNA-derived 5hmC biomarkers.

## The biology of 5hmC

**Fig. 2:**
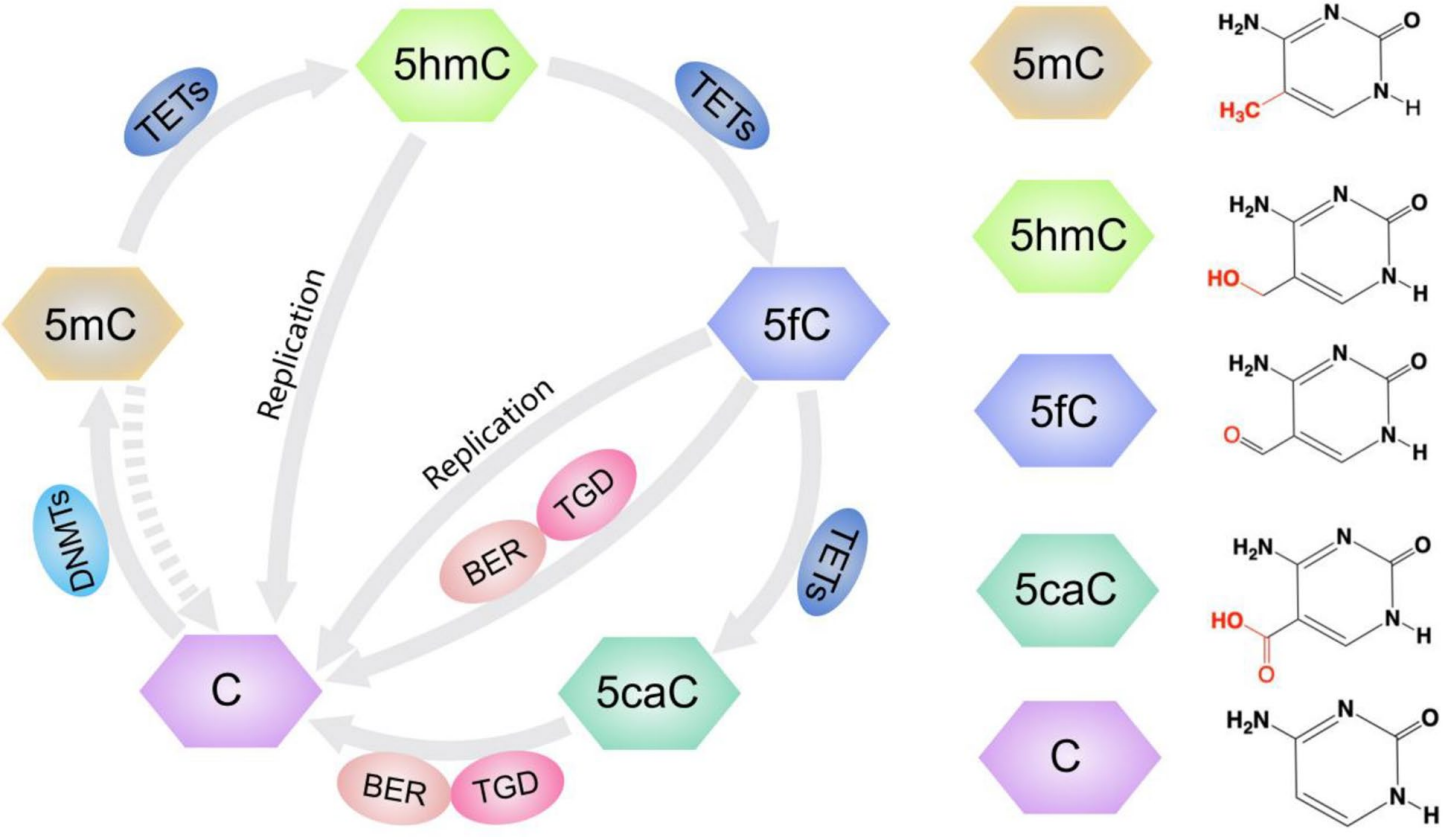
Cytosine modifications and TET-mediated oxidation pathways in epigenetic regulation. This figure illustrates the sequential oxidation of 5mC by the TET family of enzymes, converting 5mC into 5hmC, then further to 5fC, and finally to 5caC. These modifications play a crucial role in gene regulation and active DNA demethylation. The cycle also involves BER and TDG, which facilitate the removal and replacement of modified cytosines. The right panel displays the chemical structures of each cytosine modification. TET, ten-eleven translocation; C, cytosine; 5mC, 5-methylcytosine; 5hmC, 5-hydroxymethylcytosine; 5fC, 5-formylcytosine; 5caC, 5-carboxycytosine; BER, base excision repair; TDG, thymine-DNA glycosylase

Genetic and epigenetic alterations, including changes in DNA methylation patterns, are hallmarks of cancer [15]. In this context, modifications involving 5-methylcytosine (5mC), a major epigenetic mark, are prevalent in mammalian genomes and play crucial roles in gene regulation [16]. The ten-eleven translocation (TET) family of enzymes – TET1, TET2, and TET3 – catalyzes the oxidation of 5mC to 5hmC, followed by further oxidation to 5-formylcytosine (5fC) and 5-carboxylcytosine (5caC) [17] (Fig. 2). Over the past decade, studies have revealed that 5hmC, while serving as an intermediate in DNA demethylation, is enriched in transcriptionally active regions such as promoters, gene bodies, and enhancers, and may also play multiple independent roles beyond its function in the demethylation process [18–21]. Notably, certain 5hmC modifications can serve as stable epigenetic marks throughout the cell cycle, distinguishing them from other oxidized derivatives like 5fC and 5caC, which are more transient [22]. Studies have consistently demonstrated that 5hmC levels are significantly reduced in both hematologic and solid tumors compared to normal tissues [23]. However, local increases in 5hmC at specific gene regulatory elements have also been observed, highlighting its complex role in cancer biology. The dual patterns of global loss and localized enrichment of 5hmC in tumors underscore its significance in cancer initiation, development, and progression [24]. These unique epigenetic signatures make 5hmC a promising biomarker for cancer screening, diagnosis, disease monitoring, and prognosis evaluation, with the potential to improve precision in oncological research and clinical applications.

## Current sequencing approaches for 5hmC

The primary strategies for mapping the distribution and localization of 5hmC can be broadly categorized into base-resolution and enrichment-based methods. Table 1 summarizes the strengths and limitations of various 5hmC detection methods.

**Table 1:** Overview of 5hmC detection methods

Techniques	Strengths	Limitations
BS-seq [25]	Gold standard Single-base resolution	Fail to distinguish 5mC from 5hmC High input
TAB-seq [26]	Single-base resolution	DNA degradation High input High sequencing depth
oxBS-seq [27]	Single-base resolution	DNA degradation High input High sequencing depth
TAPS [28]	No DNA degradation Highly specificity	Depends on the activity of TET Low resolution
hmC-CATCH [29]	No DNA degradation Low input Low cost	PCR bias
hMe-Seal/Nano-hmC-Seal [30–32]	Highly specificity Genome-wide profiling Cost-effective Low input	Low resolution
SMRT sequencing [33]	Long read lengths Single molecule analysis	High error rate High cost High input High sequencing depth
Nanopore sequencing [34]	Ultra-long read lengths (> 1 Mb)	High error rate High cost High sequencing depth

*Abbreviations: 5hmC* 5-hydroxymethylcytosines, *5mC* 5-methylcytosine, *BS-seq* Bisulfite sequencing, *oxBS-seq* Oxidative bisulfite sequencing; *TAB-seq* TET-assisted bisulfite sequencing; *TAPS* TET-assisted pyridine borane sequencing; *SMRT* Single Molecule Real-Time

### Base-resolution methods

Base-resolution epigenomic analyses enable precise identification of modified bases within the sequenced region. Bisulfite sequencing (BS-seq) is a traditional method for profiling 5hmC modifications but cannot accurately distinguish between 5mC and 5hmC due to the indiscriminate conversion of cytosine (C) to thymine (T) during bisulfite treatment [25]. To address this limitation, modified bisulfite-based methods, such as TET-assisted bisulfite sequencing (TAB-seq) and oxidative bisulfite sequencing (oxBS), have been developed [26, 27]. The TAB-seq involves an initial protection step whereby 5hmC is enzymatically converted to glucosylated 5hmC (β-glucosyl-5hmC) through β-glucosyltransferase (βGT), and it is protected due to its modified chemical structure during the TET oxidation process. Subsequent bisulfite treatment results in the selective conversion of unmodified C and oxidized 5mC bases, while the protected 5hmC bases remain unchanged and are detected as C during sequencing analysis [26]. In the oxBS-seq process, 5hmC is selectively oxidized to 5fC by potassium perruthenate (KRuO₄), followed by bisulfite treatment. Consequently, 5hmC bases in DNA are converted to C-to-T transitions, while 5mC bases remain unconverted and are still detected as C [27]. While effective, modified bisulfite-based techniques often cause DNA degradation, which poses challenges when working with limited DNA samples or low-input material, such as cfDNA. Furthermore, these methods may suffer from inadequate bisulfite conversions, such as 5fC [24].

To overcome these drawbacks, bisulfite-free alternatives have been introduced. Techniques such as TAPS/TAPS-β, ACE-seq, and Six-letter sequencing enable more efficient analysis with minimal amounts of DNA material [28, 35–37]. Another notable bisulfite-free approach, hmC-CATCH, enables single-base 5hmC detection while minimizing DNA degradation and requiring only minimal input DNA, rendering it highly suitable for cfDNA studies [29]. However, base-resolution methods require high sequencing depth to detect low-level changes, which can significantly increase costs and limit their scalability in clinical settings.

### Enrichment-based methods

Enrichment-based approaches selectively target 5hmC-enriched regions using affinity pulldown techniques. One widely used method, 5hmC-Seal, enables genome-wide 5hmC mapping with input requirements as low as 1 ng, making it ideal for analyzing rare cell populations, tissues, or cfDNA [19]. Improved variations, such as hMe-Seal and Nano-hmC-Seal, have been developed to enhance sensitivity and specificity for cfDNA profiling [30–32]. Compared to base-resolution approaches, enrichment-based methods are more sensitive to low-abundance modifications, require less input DNA, are more cost-effective, and have higher throughput. Despite their advantages, enrichment-based methods cannot precisely localize modifications at single-base resolution, limiting their application in fine-scale mapping within the DNA fragment. Recent advancements, including Jump-seq and hmTOP-seq [38, 39], provide near-base resolution while maintaining cost-effectiveness in situations where full base-resolution is not required.

### Third-generation sequencing technologies

Third-generation sequencing platforms, such as Single Molecule Real-Time (SMRT) sequencing and Nanopore sequencing [33, 34], mark significant advancements in 5hmC profiling, enabling long-read, non-destructive sequencing of genetic and epigenetic information simultaneously, offering comprehensive insights into genome-wide patterns, including fragmentomics and 5hmC modifications [40]. While those methods hold great promise for integrating multiple layers of genomic information, challenges remain regarding their clinical feasibility. Third-generation sequencing technologies often exhibit higher error rates compared to second-generation sequencing platforms, requiring additional computational corrections [40, 41]. Moreover, their current cost per sample remains high, posing barriers to widespread adoption in routine clinical practice [42]. However, ongoing improvements in sequencing accuracy and reductions in cost may address these limitations, making these approaches more viable for translational applications.

## Cancer early detection and diagnosis

Advances in cfDNA sequencing technologies have facilitated the clinical application of cfDNA profiling for the early screening and diagnosis of cancers. Cancer specific 5hmC signatures in circulating cfDNA serve as promising epigenetic markers for distinguishing cancer patients from non-cancer individuals. These unique patterns hold significant potential for the early detection and diagnosis of various malignancies. A summary of the detection performance of cfDNA-5hmC signatures in various cancers is provided in Table 2.

**Table 2:** Quantitative performance of 5hmC signatures in circulating cfDNA in the early cancer detection

Classifier	Study design	No. of participants	Cancer types	Sequencing method	Sensitivity	Specificity	AUC
The cell-free 5hmC signatures [43]	Unknown	NSCLC (*n* = 66) and healthy individuals (*n* = 67)	Lung cancer *vs.* healthy controls	5hmC-Seal Illumina NextSeq 500	None	None	Train: 0.927 Val: 0.960
A 37-feature 5hmC model [44]	Retrospective	Lung cancer (*n* = 157) and healthy individuals (*n* = 189)	Lung cancer *vs.* healthy controls	5hmC-Seal Illumina Novaseq 6000	Val-1: 87.50% Val-2: 72.73%	Val-1: 83.87% Val-2: 80.60%	Val-1: 0.894 Val-2: 0.848
A model integrating 5hmC signals and fragment profiles [44]					Val-1: 87.50% Val-2: 83.33%	Val-1: 90.30% Val-2: 77.61%	Val-1: 0.943 Val-2: 0.864
A weighted diagnostic model comprised of 105 features (wd-score) [45]	Unknown	NSCLC (*n* = 727), SCLC (*n* = 41), precancerous lesions (*n* = 80), benign tumors (*n* = 50), and health controls (*n* = 1,092)	Lung cancer *vs.* healthy controls	5hmC-Seal Illumina NextSeq 500	Train: 84.3% Val-1: 82.5% Val-2: 76.8% Val-3: 76.6%	Train: 92.3% Val-1: 90.4% Val-2: 89.6% Val-3: 88.6%	Train: 0.961 Val-1: 0.931 Val-2: 0.902 Val-3: 0.864
6 bp-5hmC score [46]	Prospective	Participants indeterminate pulmonary nodules (*n* = 2,032), including those with malignant diseases (*n* = 1,598) and benign diseases (*n* = 434)	Lung cancer *vs.* healthy controls	hMe-Seal Illumine NovaSeq6000	Val: 67.4% Int Test: 67.9% Ext Test: 71.7%	Val: 50.0% Int Test: 50.0% Ext Test: 52.2%	Val: 0.642 Int Test: 0.642 Ext Test: 0.675
clinic-Rad(h)mC (the model established by combining clinical variables, the DL-radiomics model score, the 6 bp-5mC model score with the 6 bp-5hmC model score) [46]					Val: 81.2% Int Test: 82.8% Ext Test: 90.7%	Val: 77.4% Int Test: 81.4% Ext Test: 80.6%	Val: 0.793 Int Test: 0.821 Ext Test: 0.856
A model-based classifier [18]	Unknown	CRC (*n* = 71) and controls (*n* = 90)	Colorectal cancer *vs.* controls	Nano-hmC-Seal Illumina Hi-Seq or NextSeq 500	Val-1: 83% Val-2: 88%	Val-1: 94% Val-2: 89%	Val-1: 0.95 Val-2: 0.94
A 96-gene machine-learning model [47]	Prospective	CRC (*n* = 1,074), AA (*n* = 356), other solid tumors (*n* = 80), and non-CRC/AA controls (*n* = 1,066)	Colorectal cancer/advanced adenomas *vs.* controls	5hmC-Seal Illumina NGS	Int Val: 80.0% Ext Val: 71.3%	Int Val: 87.4% Ext Val: 83.0%	Int Val: 0.926 Ext Val: 0.872
A XGBoost method-based classifier [48]	Unknown	Esophagus cancer (*n* = 150) and healthy controls (*n* = 177)	Esophagus cancer *vs.* healthy controls	Nano-hmC-Seal Illumina NextSeq 500	Test:93.75%	Test: 85.71%	Test: 0.972
A 5hmC-based model [49]	Retrospective	ESCC (*n* = 250) and healthy individuals (*n* = 254)	ESCC *vs.* healthy controls	5hmC-Seal Illumina CN500	Int Val: 69.3% Ext Val: 74.3%	Int Val: 82.4% Ext Val: 90.7%	Int Val: 0.810 Ext Val: 0.862
An integrated model [49]					Test: 82.4%	Test: 88.2%	Test: 0.934
A regularized regression model [50]	Retrospective	PDAC (*n* = 64) and non-cancer controls (*n* = 243)	PDAC *vs.* controls	Illumina NextSeq 550	None	None	Train: 0.919 Val-1: 0.921 Val-2: 0.943
A 27-feature 5hmC model [51]	Unknown	PDAC (*n* = 61) and healthy controls (*n* = 86)	PDAC *vs.* healthy controls	hMe-Seal Illumina NovaSeq 6000	Val: 78.6% Val-1: 85.7%	Val: 100% Val-1: 93.3%	Val: 0.992 Val-1: 0.960
The 51-feature model combining 5mC and 5hmC markers [51]					Val: 93.8%	Val: 95.5%	Val: 0.997
A machine learning model [52]	Prospective	Pancreatic cancer (*n* = 234), noncancer subjects (*n* = 2,576), and non-pancreatic cancer (*n* = 1,524)	Pancreatic cancer *vs.* controls	hMe-Seal Illumina NovaSeq 6000	Train: 65.9% Val: 66.7%	Train: 97.9% Val: 96.9%	None
wd-score [53]	Prospective	HCC (*n* = 1,204), CHB or liver cirrhosis (*n* = 392), benign liver lesions (*n* = 388), healthy controls (*n* = 570)	HCC *vs.* non-HCC controls	5hmC-Seal Illumina NextSeq 500	Train: 89.6% Val-1: 82.7%	Train: 78.9% Val-1: 76.4%	Train: 0.923 Val-1: 0.884 Val-2: 0.887
HCC score [54]	Retrospective	HCC (*n* = 135), liver cirrhosis (*n* = 62) and healthy controls (*n* = 165)	HCC *vs.* non-HCC controls	hMe-Seal Illumina NovaSeq 6000	Train: 78.64% Test: 93.75%	Train: 90.91% Test: 77.78%	Train: 0.92 Test: 0.95
an HIFI model [55]	Prospective	Liver cirrhosis (*n* = 2,250), HCC (*n* = 508), and healthy controls (*n* = 476)	HCC *vs.* non-HCC controls	llumina NovaSeq 6000	None	None	Val: 0.995 Test: 0.996
PreCar Score [56]	Prospective	HCC (*n* = 510) and liver cirrhosis (*n* = 4,367)	HCC *vs.* liver cirrhosis	llumina NovaSeq 6000	Val: 93.75%	Val: 95.4%	None
A classifier model [57]	Unknown	Gastric cancer (*n* = 50) and healthy controls (*n* = 50)	Gastric cancer *vs.* healthy controls	5hmC-Seal Nova6000 plat form	Train: 88.6% Val: 73.3% Test: 81.9%	Train: 94.3% Val: 93.3% Test: 90.2%	Train: 0.96 Val: 0.87 Test: 0.90
wd-scores [58]	Prospective	Gliomas (*n* = 111) and matched healthy controls (*n* = 111)	Gliomas *vs.* healthy controls	5hmC-Seal Illumina NextSeq 500	None	None	Train: 0.87 Test: 0.83
5hmC classifier [59]	Prospective	Glioma (*n* = 291) and healthy controls (*n* = 340)	Gliomas *vs.* healthy controls	hMe-Seal Illumina NovaSeq 6000	Test: 89.2% Val: 90.1%	Test: 91.3% Val: 81.1%	Test: 0.962 Val: 0.930
wp-scores [60]	Unknown	Acute myeloid leukemia (*n* = 103) and non-cancer controls (*n* = 81)	Acute myeloid leukemia *vs.* controls	Nano-hmC-Seal NovaSeq 6000	None	None	Val: 0.981 Test: 0.946
wd-score [61]	Unknown	Acute myeloid leukemia (*n* = 115) and matched non-cancer controls (*n* = 86)	Acute myeloid leukemia *vs.* controls	Nano-hmC-Seal NovaSeq 6000	None	None	Train:1.000 Val: 0.960 Test: 0.984
A pan-cancer signature [62]	Retrospective	Bladder (*n* = 41), breast (*n* = 62), colorectal (*n* = 45), kidney (*n* = 54), lung (*n* = 57), prostate (*n* = 125) cancer and non-cancer controls (*n* = 221)	Cancer *vs.* controls	Nano-hmC-Seal NovaSeq 6000	Train: 60.6% Val: 68.6%	Train: 97.7% Val: 96.6%	None
A pan-cancer signature (53 5hmC signatures) [63]	Retrospective	LUAD (*n* = 33), 74 PDAC (*n* = 74), HCC (*n* = 132), GBM (*n* = 72) and controls (*n* = 85)	Cancer *vs.* controls	Public database	Val: 82.26% Test: 81.97%	Val: 82.35% Test: 82.35%	Val: 0.876 Test: 0.872
A pan-cancer signature (fragmentomic features and 5hmC signatures) [63]				Public database	Val: 93.44% Test: 88.52%	Val: 88.24% Test: 82.35%	Val: 0.927 Test: 0.920

*Abbreviations: NSCLC* non-small cell lung cancer, *SCLC* small cell lung cancer, *CRC* colorectal cancer, *AA* advanced adenomas, *ESCC* esophageal squamous cell carcinoma, *PDAC* pancreatic ductal adenocarcinoma, *HCC* hepatocellular carcinoma, *CHB* chronic hepatitis B virus infection, *GBM* glioblastomas, *5hmC* 5-hydroxymethylcytosines, *Int Val* internal validation, *Ext Val* external validation

### Lung cancer

Lung cancer remains the leading cause of cancer-related mortality worldwide [64]. Low-dose computed tomography (LDCT) has been implemented for the early screening of lung cancer, leading to a 31% reduction in mortality among high-risk populations [4]. However, its broader application has been limited by the low specificity [65] and the potential risks associated with radiation exposure [66]. The 2021 USPSTF guidelines emphasized the need for further research to improve LDCT screening and to identify precise biomarkers for high-risk or tumor-bearing individuals [67].

Several studies have demonstrated the potential utility of cfDNA-5hmC signatures for lung cancer detection. For instance, Zhang et al. developed a machine learning classifier based on genome-wide 5hmC profiles from plasma cfDNA to distinguish lung cancer patients from healthy controls, achieving high accuracy in both training (area under curve [AUC] = 0.93) and validation (AUC = 0.96) datasets [43]. Similarly, a 37-feature 5hmC-based model differentiated lung cancer patients from healthy individuals, achieving sensitivities of 87.50% and 72.73% and specificities of 83.87% and 80.60% in two independent validation sets [44].

To further enhance detection, a fragmentation-based model incorporating 48-features demonstrated strong performance, with AUCs of 0.93 and 0.82 across validation sets (sensitivity = 87.50% and 78.79%, specificity = 80.65% and 76.12%, respectively) [44]. Another study utilized genome-wide 5hmC profiling from 1,990 cfDNA samples to establish a weighted diagnostic model (wd-score) with 105 features, achieving an AUC range of 0.864 to 0.931 in internal and external validation sets [45]. This model significantly outperformed serum biomarkers (*p* < 0.001) and demonstrated high accuracy in detecting high-risk pulmonary nodules (AUC = 0.82) and various lung cancer subtypes [45]. Moreover, integrating 5hmC in gene bodies with fragment profiles further improved performance, achieving AUCs of 0.94 and 0.86 in two validation sets, with sensitivities of 87.50% and 83.33%, and specificities of 90.30% and 77.61%, respectively [44].

A recent multi-centric study involving 2,032 participants with indeterminate pulmonary nodules integrated clinical, radiomic, and circulating cfDNA fragmentomic features to develop a multi-omics model for predicting malignancy risk [46]. The study identified the 6 bp-5mC and 6 bp-5hmC scores—derived from 6-mer end motifs in 5mC- and 5hmC-sequencing data, respectively—as significant predictors of malignancy in multivariable logistic regression analyses. The clinic-RadmC model, integrating clinical data, deep learning-based radiomics and 5 bp-5mC scores, achieved an AUC of 0.923 on an external test set, outperforming single-omics models and those integrating only clinical data with radiomic or 5mC fragmentomic features [46]. However, incorporating 6 bp-5hmC score did not further enhance the clinic-RadmC model’s performance [46].

The exploration and validation of 5hmC signatures in cfDNA has the potential to facilitate a post-test risk assessment, thereby guiding clinical decisions in the management of indeterminate pulmonary nodules. For instance, 5hmC-based biomarkers in cfDNA may offer a solution for nodules detected during annual screenings, which are often too small to be amenable to current biopsy techniques. In particular, 5hmC modifications in cfDNA may prove beneficial in cases where patients with nodules require closer surveillance or a decision regarding biopsy. When biopsy is inaccessible (e.g., near heart or vessels), 5hmC signatures in cfDNA could provide a complement for diagnostic purpose. Integration of multi-omics/multi-modal data could improve the predictive performance but still needs to be further explored. Collectively, these studies highlight the potential of 5hmC biomarkers in cfDNA to serve as a non-invasive, blood-based tool for the early detection and diagnosis of lung cancer, offering radiation-free alternatives to current screening methods.

### Colorectal cancer

Colorectal cancer (CRC) is a prevalent gastrointestinal tumor, ranking fourth in incidence and second in mortality in the United States [64]. Early detection of CRC extends the window for curative treatment and significantly reduces cancer-specific mortality [68]. Current screening methods, including colonoscopy and stool-based tests like fecal occult blood testing (gFOBT), fecal immunochemical testing (FIT), and multitargeted stool DNA (MT-sDNA), are commonly used for CRC screening [69]. However, the limited sensitivity of stool-based tests often leads to false-negative results in early-stage CRC [6, 69]. Furthermore, the invasive nature of endoscopy and its potential complications frequently hinder patient adherence [6, 69]. This highlights the urgent need for non-invasive, highly accurate, blood-based techniques for early CRC detection.

Alterations in 5hmC modifications provide promising biomarkers for CRC, offering distinct clinical evidence, particularly in cases with or without a history of adenoma [70]. Plasma cfDNA-5hmC signatures have been extensively investigated for CRC detection, with genes such as *LCN2*, *LRG1*, *S100P*, *TACSTD2*, and *TLR4* identified as potential non-invasive markers [71, 72]. Additionally, higher levels of *ZW10* 5hmC were observed in both adenoma and CRC groups (*n* = 30 each) compared to the controls (*n* = 31), achieving an AUC of 0.75 in the CRC group [73]. However, as these findings are based on small cohorts, further validation in larger populations is essential.

Exploration of cfDNA-5hmC signatures generated by multiple markers has shown promise in enhancing the accuracy of CRC detection. Prior research has demonstrated that 5hmC-based biomarkers in circulating cfDNA can predict CRC and outperform traditional tumor biomarkers [18, 74]. For example, a prospective case–control study (NCT03676075) utilized cfDNA-5hmC signatures to distinguish stage I-III CRC and advanced adenomas from controls. This study involved 2,576 participants, including patients with CRC (*n* = 1,074), advanced adenomas (*n* = 356), other solid tumors (*n* = 80), and non-colorectal carcinoma/advanced adenomas controls (*n* = 1,066). The proposed weighted diagnostic scores (wd-scores), which comprise a 96-gene model, achieved high accuracy with AUCs of 0.93 in internal validation and 0.87 in external validation set [47]. Furthermore, the wd-score demonstrated superior accuracy and sensitivity compared to carcinoembryonic antigen (CEA), a commonly used protein biomarker, particularly in identifying high-risk adenomas and early-stage CRC [47]. Another study utilizing decades-old plasma samples (included 201 CRC cases and 401 controls) from the PLCO Cancer Screening Trial identified 5hmC-modified cfDNA biomarkers capable of detecting CRC up to 36 months before clinical diagnosis, with AUCs of 0.77 and 0.73 in the training and validation sets, respectively [75]. This 5hmC-based predictive model significantly outperformed traditional risk factors, such as age and BMI, and demonstrated consistent performance across sex and race/ethnicity, highlighting its potential for early, non-invasive CRC detection.

These findings highlight the potential of genome-wide 5hmC mapping in cfDNA as a non-invasive and highly accurate biomarker for the early detection of CRC and high-risk advanced adenomas. The simplicity and non-invasive nature of this blood-based test could significantly improve patient adherence to CRC screening compared to stool-based tests or colonoscopy. However, further longitudinal and prospective studies are required to validate the efficacy and cost-effectiveness of 5hmC signatures in comparison to standard CRC screening methods, particularly in larger and more diverse populations.

### Esophageal cancer

Esophageal cancer (EC) is the seventh most common cancer in terms of incidence and ranks sixth as a cause of cancer-related mortality worldwide [76]. Given the aggressive nature of EC and the prevalence of advanced-stage diagnosis, early detection of EC is crucial, especially in high-prevalence regions. Currently, upper endoscopy, particularly Lugol iodine chromoendoscopy, is the primary method for EC detection [77, 78]. However, the complexity of the procedure and the low compliance, similar to challenges faced with colonoscopy, limit its feasibility for mass screening [79]. This highlights the need for non-invasive, high-compliance early detection methods to enable timely intervention and improve patient outcomes.

Emerging evidence has highlighted the potential of 5hmC modifications in cfDNA for early EC detection. A study involving 150 newly diagnosed EC patients and 177 healthy individuals identified robust 5hmC-based signatures capable of differentiating EC patients from healthy controls, with an average AUC of 0.95 in the testing set and 0.96 in the validation set [48]. Another recent study investigated and validated cfDNA-5hmC signatures in patients with esophageal squamous cell carcinoma (ESCC). This study employed an independent dataset comprising 150 ESCC patients and 183 healthy individuals for external validation. The resulting diagnostic model, based on cfDNA-5hmC signatures, achieved an AUC of 0.81 in the internal validation set and 0.86 in the external validation set [49].

Integrating multi-omics data has shown promise in improving the performance of early detection of various cancers [80, 81]. For instance, an integrated model combining low-pass whole-genome sequencing (WGS) and 5hmC biomarkers demonstrated improved diagnostic accuracy for early ESCC, achieving an AUC of 0.93, a sensitivity of 82.40%, a specificity of 88.20%, and an overall accuracy of 0.843 [49]. These results suggest that multi-omics approaches could enhance the precision of non-invasive screening tools.

These findings collectively point to 5hmC profiling in cfDNA as a non-invasive, convenient method for EC detection, offering the potential for early diagnosis. Currently, integration of multi-omics and/or multi-modal detection represents a promising approach for the non-invasive early diagnosis of various types of cancer [51, 80–82]. As aforementioned, the utilization and combination of 5hmC and WGS data efficiently differentiated very early ESCC from controls in the two cohorts [49]. Therefore, further research should focus on integrating 5hmC epigenomic signals with multi-omics and/or multi-modal data to refine early detection models for different subtypes and stages of EC. Moreover, incorporating known risk factors [83], such as alcohol consumption, body mass index, and human papillomavirus, could further improve the specificity and sensitivity of EC screening programs.

### Pancreatic cancer

Pancreatic cancer is a highly lethal malignant tumor characterized by an occult onset, low rates of early diagnosis, rapid progression, and poor prognosis [84, 85]. Currently, effective serological biomarkers for the early detection of pancreatic cancer remain elusive. Carbohydrate antigen 19–9 (CA19-9), the primary biomarker used for early detection and diagnosis, is not recommended as a standalone screening tool, according to ESMO clinical guidelines [86]. Similarly, CEA, another commonly used biomarker [87], demonstrates lower sensitivity and specificity compared to CA19-9. These limitations highlight the urgent need for novel biomarkers with improved diagnostic and monitoring performance for pancreatic cancer.

The potential of 5hmC markers in cfDNA to differentiate pancreatic cancer from healthy individuals has been reported in previous studies. Distinct 5hmC hydroxymethylation patterns between pancreatic ductal adenocarcinoma (PDAC) and non-cancer samples have been identified, particularly in genes associated with pancreas development and function (e.g., *GATA4*, *GATA6*, *PROX1*, *ONECUT1*, *MEIS2*) and cancer pathogenesis (e.g., *YAP1*, *TEAD1*, *PROX1*, *IGF1*) [50]. These genes were found to be enriched in targets of transcription factors implicated in PDAC oncogenesis and metastasis, suggesting that 5hmC profiling captures biologically relevant signals in plasma [50]. Regularized logistic regression models based on the most variable 5hmC densities exhibited excellent diagnostic accuracy, with AUCs of 0.92 and 0.94 in two independent test sets [50].

A subsequent model incorporating 27 candidate 5hmC features showed exceptional performance in two validation cohorts, achieving AUCs of 0.99 and 0.96, sensitivities of 78.6% and 85.7%, and specificities of 100% and 93.3%, respectively [51]. This model effectively distinguished stage I from stage II–IV PDAC patients, highlighting its utility for early detection [51]. Notably, a recent machine learning model leveraging 5hmC differential profiling and genomic features from 4,334 participants achieved a sensitivity of 68.30% for early-stage pancreatic cancer and an overall specificity of 96.90%, outperforming CA19-9 in early-stage detection [52]. Additionally, combining 5mC and 5hmC signatures further improved the prediction sensitivity for PDAC [51].

These findings underscore the potential of 5hmC biomarkers in cfDNA as a non-invasive tool for the early detection and diagnosis of pancreatic cancer. Furthermore, 5hmC profiling has the potential to identify individuals with intraductal papillary mucinous neoplasms (IPMNs)—recognized as precursor lesions of pancreatic cancer—especially those with moderate-to-high dysplasia [52]. Despite these advances, large-scale and prospective studies are necessary to validate the diagnostic and monitoring capabilities of 5hmC signatures, particularly in early-stage pancreatic cancer and IPMNs, to fully integrate this approach into clinical practice.

### Hepatocellular carcinoma

Hepatocellular carcinoma (HCC) is the most common subtype of primary liver cancer, contributing substantially to the global cancer burden due to its high incidence and mortality rates [88]. Current surveillance strategies recommended by the European Association for the Study of the Liver (EASL) and the American Association for the Study of Liver Diseases (AASLD) primarily include ultrasound with or without alpha-fetoprotein (AFP) testing for high-risk populations [89, 90]. However, these methods are hampered by limited sensitivity, particularly in detecting early-stage HCC [91]. This highlights the pressing need for advanced diagnostic tools with greater accuracy and reliability.

Recent advances in 5hmC-based cfDNA profiling have demonstrated great promise in addressing this challenge. Our multicenter study involving 1,204 HCC patients, 392 patients with chronic hepatitis B virus infection or liver cirrhosis, and 958 healthy individuals and benign liver lesion cases, led to the development of a 32-gene diagnostic model (wd-score) [53]. This model accurately distinguished early-stage HCC from non-HCC subjects and from high-risk individuals with chronic hepatitis B virus infection or liver cirrhosis, achieving AUCs of 0.884 and 0.846 in the validation cohort, respectively. Notably, the wd-score outperformed AFP in diagnostic performance [53]. Serum AFP has been the primary diagnostic modality for HCC, but AFP-based diagnostic performance is still far from satisfactory [92]. The elevation levels of AFP are unclear in approximately 80% of small HCCs and early-stage tumors [92]. It also lacks diagnostic specificity, particularly in those with cirrhosis and chronic hepatitis, which may result in AFP elevation [93]. Therefore, the proposed wd-score could complement AFP in HCC diagnosis. Additionally, when combined with tumor markers such as AFP and des-gamma-carboxy prothrombin (DCP), the wd-score further improved diagnostic accuracy, yielding AUCs of 0.920 in the training set and 0.950 in the testing set [54].

Innovative approaches have also explored integrating 5hmC profiling with other genomic features. For instance, the HIFI model, which incorporates 5hmC, motif, fragmentation, and nucleosome footprint data, achieved remarkable sensitivity (95.42%) and specificity (97.83%) for differentiating HCC from high-risk liver cirrhosis patients in an independent test set [55]. It also demonstrated significantly superior differentiation performance between HCC and liver cirrhosis compared to AFP, as evidenced by the AUCs of 0.996 and 0.826 in the independent cohort, respectively [55]. Furthermore, combining copy number variation (CNV) data with these genomic features significantly enhanced the sensitivity for detecting early-stage and very early-stage HCC compared to ultrasound (51.32% *vs.* 23.68%, *p* < 0.01) [56].

In summary, 5hmC profiling in cfDNA represents a transformative advancement in HCC diagnostics, particularly for early detection. Future research should prioritize longitudinal studies to track changes in cfDNA-5hmC signatures over time, enabling earlier detection of HCC recurrence or progression compared to conventional imaging and serological methods. Additionally, efforts should focus on integrating multi-omics data, such as radiomics, transcriptomics, and proteomics, with 5hmC signatures to enhance diagnostic accuracy and discover novel biomarkers for early-stage HCC. Furthermore, future studies should explore the feasibility of implementing 5hmC-based biomarkers in large-scale, diverse cohorts to validate their utility and assess cost-effectiveness in routine clinical practice. These investigations have the potential to transform HCC management and reduce disease-related mortality.

### Glioma

Glioma, the most prevalent type of intracranial tumor in adults, is often associated with a poor prognosis [94]. Early detection of glioma provides significant clinical benefits, but current diagnostic approaches are primarily reliant on radiological examinations, which are typically performed after the onset of neurological symptoms [95]. Although tumor biopsy remains the gold standard for diagnosis [95], it carries potential risks, particularly when tumors are located in anatomically sensitive regions. Consequently, there is a pressing need for non-invasive diagnostic methods to complement existing strategies and reduce the risks associated with invasive procedures.

Emerging evidence suggests that 5hmC modifications in cfDNA may enable the early detection of gliomas and provide a valuable adjunct to current diagnostic modalities. Using the highly sensitive 5hmC-Seal technique, a study developed multi-feature diagnostic models for gliomas through an integrative analysis of genome-wide 5hmC profiles in cfDNA samples. These 5hmC-based models successfully differentiated healthy individuals from patients with gliomas (AUC = 0.84), glioblastomas (AUC = 0.84), and WHO grade II–III gliomas (AUC = 0.86) [58]. Another study introduced the GDScore, a 5hmC-based diagnostic model that demonstrated high performance in distinguishing gliomas (AUC = 0.93), glioblastomas (AUC = 0.92), and lower-grade gliomas (AUC = 0.94) in validation sets [96]. Furthermore, 5hmC-based cfDNA profiles generated using the hMe-Seal method achieved exceptional accuracy, with an AUC of 0.96 in the test set and 0.94 in an independent validation set, effectively differentiating glioma patients from controls across 409 samples [59].

The mutational status of isocitrate dehydrogenase (*IDH*) genes serves as a key molecular marker for distinguishing two biologically and clinically distinct subsets of gliomas [97]. 5hmC signals in plasma cfDNA have shown promise in predicting *IDH* mutational status, achieving an AUC of 0.82 in the test set and 0.71 in the independent validation set [59]. Notably, the GDScore achieved superior performance in differentiating *IDH* wild-type gliomas (AUC = 0.93) and *IDH* mutant gliomas (AUC = 0.94) from healthy controls in the validation set [96]. Additionally, combining 5hmC biomarkers with *IDH1* mutation status enhanced differentiation between glioblastomas (GBM) from WHO II-III gliomas [58].

The application of 5hmC profiling in cfDNA offers significant value for gliomas diagnosis, particularly when traditional biopsy is contraindicated, such as in cases involving deep brain lesions or patients who are elderly, frail, or have significant comorbidities. Future studies should aim to expand the spectrum of brain diseases evaluated through 5hmC-based cfDNA profiling to better assess its sensitivity and specificity across diverse conditions. Additionally, integrating 5hmC biomarkers with other molecular and imaging data may further refine diagnostic performance, enabling earlier detection and improved stratification of glioma subtypes. Such advancements could pave the way for more personalized and non-invasive approaches to glioma diagnosis and management.

### Hematological malignancies

Hematologic malignancies, originating from the bone marrow and lymph nodes, can affect individuals of all age groups [98]. They are classified into several types, such as lymphomas, acute and chronic leukemia, and multiple myeloma (MM) [99]. Molecular testing has become an integral part of the diagnosis, subclassification, prognosis, and minimal residual disease (MRD; also known as measurable or molecular residual disease) assessment for these malignancies [100]. Epigenetic aberrations, frequently observed in hematological cancers, are thought to play a pivotal role in their initiation and progression [101].

Recent advances in 5hmC profiling have highlighted its potential as a non-invasive diagnostic tool for hematological malignancies. Using the highly sensitive and robust nano-5hmC-Seal technology, a genome-wide analysis of 5hmC distribution was performed on 239 plasma cfDNA samples from 103 acute myeloid leukemia (AML) patients and 81 non-cancer controls. A diagnostic model based on 5hmC signatures achieved outstanding accuracy, with an AUC of 0.98 in the validation set and 0.95 in the test set [60]. Similarly, another study confirmed the diagnostic potential of cfDNA-5hmC signatures for AML detection, reporting AUCs of 1.00, 0.96, and 0.98 in the training, validation, and testing cohorts, respectively [61].

In diffuse large B-cell lymphoma (DLBCL), 5hmC profiles derived from plasma cfDNA using hmC-CATCH, revealed robust cancer-associated features capable of distinguishing DLBCL patients from healthy individuals. These profiles also demonstrated the ability to differentiate between primary tumor sites, emphasizing the diagnostic versatility of 5hmC biomarkers in cfDNA [102]. Furthermore, a four-gene panel comprising *CNN2*, *HMG20B*, *ACRBP*, and *IZUMO1* successfully distinguished DLBCL from follicular lymphoma (FL), achieving an AUC of 0.89 in the testing set, regardless of the cell-of-origin or stage of DLBCL [103]. These findings suggest that the 5hmC markers derived from cfDNA hold great promise as minimally invasive biomarkers for diagnosing DLBCL, identifying primary tumor sites, and aiding in subtype classification.

Despite these advances, larger-scale and prospective studies are essential to validate the diagnostic capabilities of cfDNA-derived 5hmC biomarkers in hematologic malignancies. Currently, histological morphological analysis, immunohistochemical examination, flow cytometry, genetics, and molecular pathology analysis are the primary modalities to determine tumor subtypes in hematologic malignancies. Exploration of clinical utility of 5hmC biomarkers in subtype classification provides a clinically convenient alternative for precise tumor diagnosis. Future research should also explore their potential roles in early screening and monitoring disease progression across multiple disease spectrums, which could further enhance the precision and effectiveness of hematological cancer management.

### Pan-cancer detection

As discussed above, 5hmC signatures in cfDNA have demonstrated clinical value for detecting various cancer types. However, their potential for pan-cancer detection remains an emerging area of study. One investigation utilized genome-wide 5hmC profiling in cfDNA from 384 patients with bladder, breast, colorectal, kidney, lung, or prostate cancer and 221 controls, and randomly split 180 cancer samples at diagnosis and 70 controls into a training set and a validation set in a 6:4 ratio. Elastic net regularization on a multinomial logistic regression model was applied to identify a plasma cfDNA 5hmC signature for cancer origin classification. Consequently, a pan-cancer signature detected all six cancers with a sensitivity of 68.6% and a specificity of 96.6%, while cancer–specific signatures achieved a sensitivity of 80.0% for breast cancer, 88.9% for kidney cancer, 94.1% for lung cancer and 96.4% for prostate cancer [62]. Another study analyzed 5hmC sequencing data from 33 lung adenocarcinoma, 74 PDAC, 132 HCC, 72 GBM, and 85 control samples, and samples were randomly split into training, validation, and test sets with a ratio of 6:2:2, respectively. Random forest machine learning was used to develop pan-cancer classifiers. As a result, a 5hmC-based classifier using 53 5hmC signatures achieved an AUC of 0.88 in the validation set (sensitivity = 82.26%, specificity = 82.35%) and 0.87 in the test set (sensitivity = 81.97%, specificity = 82.35%) for cancer detection [63]. Moreover, an integrated model combining fragmentomic features with 5hmC signatures further improved detection performance, achieving an AUC of 0.93 in the validation set (sensitivity = 93.44%, specificity = 88.24%) and 0.92 in the test set (sensitivity = 88.52%, specificity = 82.35%) [63]. The integrated model also achieved high performance for each cancer detection, with AUCs of 0.95 for GBM, 0.92 for HCC, 0.88 for PDAC, and 0.76 for lung adenocarcinoma [63].

Non-invasive biomarkers such as 5hmC modifications in cfDNA are valuable in clinical settings, as they enable tumor detection and diagnosis using easily accessible clinical and laboratory data, particularly in individuals at high risk. This approach is not only practical but can also be efficiently scaled for implementation in large-scale screening programs. Collectively, cfDNA-derived 5hmC profiling is a promising biomarker for pan-cancer detection, contributing significantly to advancements in precision oncology.

## Treatment monitoring and efficacy response assessment

Radiological imaging of tumor diameter dynamics is the standard approach for assessing therapeutic response in solid tumors. However, these criteria often fail to detect disease response early and require substantial time to reveal significant changes [104, 105]. Identifying patients at high risk for resistance or progression before treatment initiation is critical for optimizing treatment decisions. Therefore, developing novel, non-invasive technologies that predict treatment effectiveness and identify individuals who are more likely to respond positively to treatment is urgently needed. Liquid biopsy, integrated into precision medicine, shows great potential for treatment selection, monitoring, and response assessment [106–108].

5hmC biomarkers in cfDNA are emerging as promising indicators of treatment response. For example, a novel multivariate model incorporating clinicopathologic data and a cfDNA-derived, 5hmC-modified gene, *OSGEPL1*, was developed to predict platinum-based chemotherapy response in intermediate-sensitive high-grade serous ovarian cancer. The model successfully stratified intermediate-sensitive patients into resistant- and sensitive-like groups [109]. Additionally, 5hmC levels are positively correlated with the expression of chemokines and programmed death-ligand 1 (PD-L1) in tumor cells, as well as the infiltration of CD3^+^ T cells, CD8^+^ T cells, and CD56^+^ NK cells. These levels were also linked to response rates for anti-PD-1/PD-L1 therapies, suggesting 5hmC could predict antitumor immunity and improve immunotherapy effectiveness [110].

In lung cancer, a 16-gene 5hmC signature (wp-scores) was developed to predict immune checkpoint inhibitor (ICI) response. Low wp-scores were significantly associated with longer progression-free survival and higher objective response rates compared to high wp-scores in ICI-treated patients [111]. In addition, wp-scores outperformed tumor PD-L1 expression in predicting ICI treatment response [111]. Dynamic changes in longitudinal 5hmC-based cfDNA profiles in non-small-cell lung cancer (NSCLC) patients undergoing anti-PD-1 therapy revealed distinct response patterns: responders exhibited increased 5hmC levels over immune activation genes (e.g., IFN-γ, IFN-α, TNF-α signaling), while non-responders showed increased 5hmC levels over epithelial-to-mesenchymal transition genes [112].

Establishing accurate markers for MRD detection is crucial for optimizing patient management and therapeutic decisions in AML [113, 114]. The wd-score, derived from a 5hmC signature, was significantly elevated in patients with MRD compared to those without, as assessed by the cfDNA 5hmC method (*p* = 2.0 × 10^–7^). This method demonstrated a concordance of 85.70% in MRD-positive samples, highlighting its reliability [61]. In DLBCL, genome-wide 5hmC profiles in plasma cfDNA from 86 patients treated with R-CHOP chemotherapy were obtained prior to therapy. A 5hmC-based logistic regression model, incorporating 13 5hmC markers, effectively predicted treatment responders and non-responders in the validation cohort, achieving an AUC of 0.78. Notably, the 5hmC markers outperformed traditional clinical indicators such as lactate dehydrogenase (LDH) levels and disease stage [115]. A machine learning model incorporating an 11-gene 5hmC signature was developed to predict treatment responses to azacitidine epigenetic priming followed by high-dose cytarabine and mitoxantrone. This model achieved an AUC of 0.86 [116]. Combining cytogenetic and pathogenic information with 5hmC signatures further enhanced prediction performance for hematologic malignancies.

Dynamic changes in 5hmC levels provide a valuable surrogate indicator for assessing solid tumor treatment responses. For example, as mentioned above, 5hmC modifications in cfDNA could be a promising indicator of MRD, which can be leveraged to predict patient response to cancer therapeutics [61]. Moreover, 5hmC signatures in cfDNA enable repeated, non-invasive testing over multiple time points, which facilitates real-time monitoring of dynamic changes of treatment response and provides a basis for timely adjustment of treatment plans. Additionally, it is especially beneficial in cases where tissue biopsies are suboptimal or inaccessible, or when resistance is suspected. Although the proposed Liquid Biopsy Response Evaluation Criteria in Solid Tumors (LB-RECIST) aims to standardize response assessment [117], clinical, biological, and regulatory challenges must be addressed before broader adoption. Future studies should explore optimal assessment timepoints, the influence of cfDNA-derived 5hmC biomarkers on therapy adjustments, and the feasibility of integrating these biomarkers into clinical practice.

## Risk stratifies and prognosis assessment

One major challenge is identifying patients who are likely to derive survival benefits from specific treatments. While tumor markers, imaging-based modalities, and pathological biomarkers are widely used as prognostic indicators, their accuracy remains limited due to tumor heterogeneity [118]. In this context, 5hmC modifications in cfDNA have shown potential for early cancer diagnosis, prognostic risk evaluation, and clinical decision-making.

In HCC, a model integrating tumor biomarkers with 5hmC signatures showed high capacity to predict recurrence risk in patients who underwent surgical resection [54]. This model served as an independent indicator for recurrence-free survival (*p* = 0.00865) and overall survival (*p* = 0.000739), and exhibited a positive correlation between HCC scores and dynamic tumor burden during follow-up [54]. Similarly, in lung cancer, a 5hmC signature (wp-score) was strongly predictive of overall survival, with low wp-scores being significantly associated with longer overall survival compared to high wp-scores in a validation cohort (*p* = 0.00059; HR [95% CI]: 0.22 [0.09–0.57]) [119]. The predictive performance of wp-scores outperformed clinical factors, such as age, sex, smoking, and TNM stage, and integrating the wp-score with other clinical variables further enhanced its prognostic utility [119].

In neuroblastoma, a model leveraging 5hmC deposition levels of 347 genes accurately predicted metastatic burden (high/moderate *vs.* low/no) in children, achieving an AUC of 0.77, a sensitivity of 70.00%, and a specificity of 89.50% [32]. Moreover, 5hmC profiles generated from 1,242 neuronal pathway genes were associated with relapse risk in low/no metastatic burden clusters, with a sensitivity of 65.00% and a specificity of 75.60% [32]. Adrenergic and mesenchymal-specific 5hmC signatures were also linked to metastatic burden, while bivalent genes identified through 5hmC-based cfDNA profiling were independently associated with poor outcomes in high-risk neuroblastoma patients [120, 121]. Collectively, these findings suggest that 5hmC patterns in neuroblastoma could serve as biomarkers for assessing metastatic burden, evaluating treatment response, and predicting outcomes.

In AML, wp-scores were significantly associated with overall survival, regardless of whether patients received hematopoietic stem cell transplantation (HSCT) (*p* = 0.00719 for HSCT recipients; *p* = 9.34e − 05 for non-recipients). High wp-scores correlated with shorter event-free survival (EFS) (*p* = 0.0234) and wp-scores were elevated in post-HSCT patients who later relapsed compared to those in complete remission (*p* = 6.19e − 6) [60]. Furthermore, 5hmC levels in genomic regions marked by H3K4me3 were used to classify AML samples into three distinct clusters associated with leukemia burden and survival [122]. MRD assessment using cfDNA-5hmC signatures was significantly correlated with outcomes; patients with high wd-scores (indicative of MRD) had shorter relapse-free survival (median 10.6 months *vs.* 31.6 months, *p* = 0.0092) [61]. Another study showed that patients with predicted *IDH* mutant gliomas displayed significantly better outcomes (*p* = 0.01) [59]. The combination of 5hmC biomarkers with *IDH1* mutations further enhanced the ability to distinguish glioblastomas from lower-grade gliomas, supporting the value of 5hmC signatures in prognosis stratification [58].

Liquid biopsies using cfDNA-5hmC signatures offer a paradigm shift toward more precise and individualized cancer management by complementing traditional methods. While 5hmC-based profiling cannot yet replace imaging or pathological evaluations for locating recurrence or metastasis, it offers valuable complementary insights for prognostic assessment. Identifying patients with high recurrence or mortality risks using 5hmC signatures can guide the frequency of follow-up examinations and optimize surveillance strategies. Longitudinal tracking of 5hmC levels could enable the early detection of recurrence before radiological confirmation, allowing for timely intervention.

## Challenges in translating cfDNA-5hmC signatures towards clinical practice

**Fig. 3:**
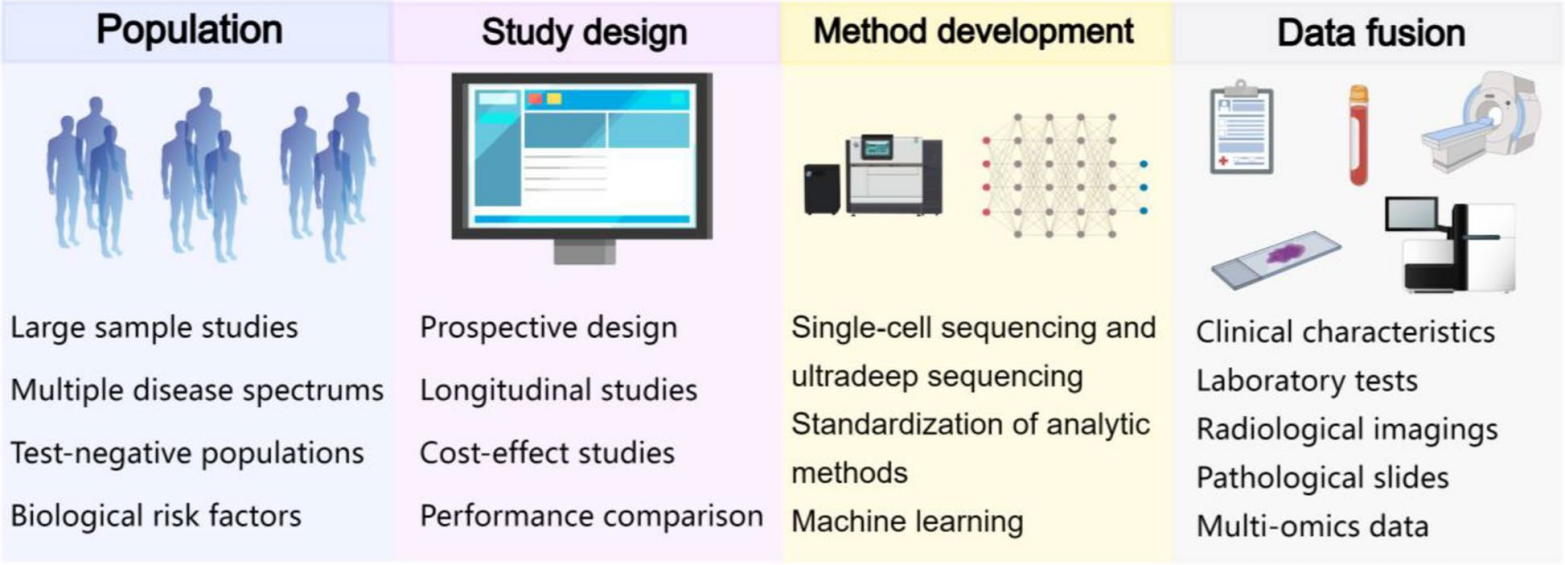
Key considerations for translating cfDNA-5hmC signatures into clinical applications. Successful clinical implementation of cfDNA-5hmC signatures requires addressing key challenges across multiple domains. Population considerations include large-scale, diverse cohorts, incorporation of test-negative populations, and biological risk factors to improve generalizability. Study design must incorporate prospective, longitudinal, and cost-effectiveness studies to validate real-world clinical utility. Method development focuses on improving sequencing technologies, standardization, and machine learning applications to enhance detection accuracy. Data fusion integrates clinical characteristics, laboratory tests, imaging, pathology, and multi-omics data, optimizing cfDNA-derived 5hmC modifications’ role in early cancer detection, prognosis, and treatment monitoring. cfDNA, cell-free DNA; 5hmC, 5-hydroxymethylcytosine; cfDNA-5hmC signatures, 5hmC signatures in circulating cfDNA

Despite promising findings, significant challenges must be addressed before 5hmC biomarkers in cfDNA can be effectively integrated into clinical practice (Fig. 3). Most current studies rely on retrospective samples and focus on evaluating the diagnostic and prognostic value of cfDNA-5hmC signatures in specific cancer types. However, limited sample sizes and the single type/subtype of cancers may not fully capture the complexities of real-world clinical settings. To bridge this gap, large-scale, multicenter, and well-designed prospective studies are urgently needed to validate the diagnostic accuracy, clinical utility, and cost-effectiveness of cfDNA-5hmC signatures across diverse cancer types and populations.

Biological factors such as age, sex, and ethnicity can influence cfDNA release and 5hmC patterns [123–125], necessitating the inclusion of diverse patient cohorts in future studies. Genetic and epigenetic variations across ethnicities may affect the clinical performance of 5hmC-based biomarkers [123], underscoring the need for stratified analyses to account for these variables. Inclusive study designs will help ensure that the resulting diagnostic models are robust and applicable to a broad range of populations.

The clinical translation of 5hmC patterns in cfDNA is further hindered by the lack of standardized protocols for sample collection, quality control, and analysis methods [126]. Variations in pre-analytical procedures, such as differences in blood collection, storage conditions, and DNA extraction methods, can significantly impact cfDNA quality and downstream results [126, 127]. Efforts to establish standardized quality control measures—such as those undertaken by the NCI Liquid Biopsy Consortium and European Liquid Biopsy Society (ELBS)—must be expanded to specifically address the unique requirements of 5hmC-based cfDNA profiling. International collaboration is vital to harmonize these protocols and ensure reproducibility and reliability across laboratories and studies.

Additionally, detecting low-abundance cfDNA fragments originating from rare tumor sub-populations remains a technical challenge. Advances in single-cell and ultra-deep sequencing technologies, as well as the development of machine learning algorithms, may enhance detection sensitivity and precision [125, 127, 128]. These innovations could also reduce sequencing costs, making cfDNA-derived 5hmC assays more accessible for routine clinical use.

Current evidence highlights the promising diagnostic utility of 5mC, fragmentomics, and other cfDNA features in cancer detection [44, 46, 51, 63]. As such, a systematic comparison of 5hmC signatures with these established biomarkers emerges as a valuable direction. Additionally, combining 5hmC biomarkers with other data modalities—such as clinical features, imaging characteristics, pathological findings, and multi-omics datasets—holds the potential to enhance early detection, therapeutic monitoring, and personalized disease management. For example, integrating cfDNA profiles with imaging data could improve early cancer detection [129, 130], while combining them with genomic and transcriptomic data could yield more accurate subtype classifications [131]. The performance improvement of multi-omics models depends on the high-quality and unbiased datasets, as well as proper integration methods and computational algorithms [132]. Registration and/or fusion of multi-omics data, security, privacy, standardization, and data access, storage, and sharing remain the major challenges. Collaborative data-sharing initiatives and comprehensive databases such as CFEA [133] and PETCH-BD [134] are valuable resources that can facilitate integrated analyses and foster innovation.

Looking ahead, developing a unified cfDNA-5hmC signature for pan-cancer detection represents an exciting direction. However, as aforementioned, current investigations into 5hmC modifications-based pan-cancer detection and tissue-of-origin determination predominantly rely on public datasets characterized by limited tumor sample sizes, restricted tumor type diversity, and a lack of independent validation. To reach this coverage, expanding tumor sample types, determining optimized cutoff values for different cancer types, and validating these signatures in large-scale, multicentric, longitudinal studies are critical next steps. It is important to note that single-cell analysis has been demonstrated to reveal epigenetic differences between different cells within a tumor, thus facilitating a more comprehensive understanding of tumor heterogeneity [135]. 5hmC analysis at the single-cell level has been shown to minimize the loss of target DNA in single cells, yield higher-resolution epigenetic information, and achieve more coverage [136, 137], all of which are crucial for determining the tissue-of-origin and classification. This, in turn, contributes to more accurate pan-cancer early detection and treatment methods. Additionally, evaluating the lower detection limits of cfDNA and incorporating findings into clinical guidelines will help address practical challenges in implementation.

## Conclusions and perspectives

**Fig. 4:**
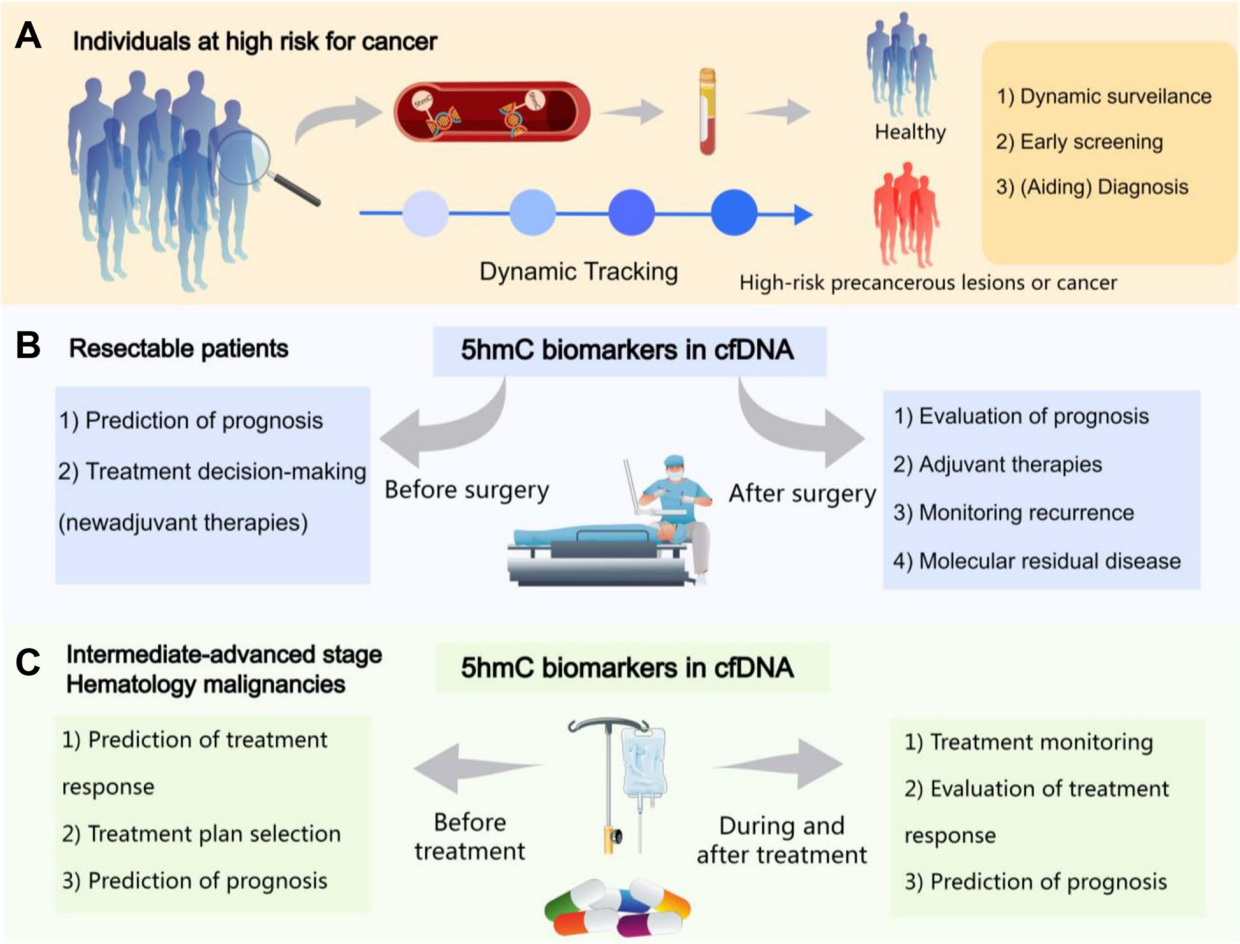
Clinical applications of 5hmC biomarkers in circulating cfDNA for cancer detection, treatment monitoring, and prognosis assessment. This figure illustrates key applications of 5hmC biomarkers in cfDNA across different clinical scenarios. **A** In the individuals at high-risk for cancer, cfDNA-5hmC signatures enables dynamic surveillance, early screening, and aiding in cancer diagnosis. **B** For resectable tumors, 5hmC biomarkers can assist in prognosis prediction, treatment decision-making (such as neoadjuvant therapies), and monitoring for recurrence post-surgery. **C** In hematologic malignancies and advanced-stage solid tumors, 5hmC profiling facilitates real-time treatment monitoring, evaluation of therapy response, and prognosis prediction. The non-invasive nature of cfDNA-5hmC signatures makes it a promising tool for personalized cancer management. 5hmC, 5-hydroxymethylcytosine; cfDNA, cell-free DNA; cfDNA-5hmC signatures, 5hmC signatures in circulating cfDNA

Reliable and accessible cancer biomarkers are essential for advancing precision medicine and improving outcomes for cancer patients. Integrating 5hmC profiling in circulating cfDNA shows significant promise in streamlining the clinical pathway from diagnosis to treatment. Specifically, 5hmC biomarkers in cfDNA has demonstrated potential for non-invasive tumor surveillance and diagnosis, particularly in populations at high risk for cancer (Fig. 4).

Although 5hmC biomarkers cannot completely replace traditional screening methods, integrating them with existing diagnostic tools could significantly enhance diagnostic accuracy. For instance, current tumor staging relies heavily on preoperative imaging and postoperative pathology to guide neoadjuvant or adjuvant therapies for resectable solid tumors [138, 139]. The unique ability of cfDNA-derived 5hmC modifications to capture intra- and inter-tumor heterogeneity makes them invaluable for identifying patients at high risk of recurrence and facilitating personalized treatment plans.

In the case of unresectable solid tumors or hematologic malignancies, cfDNA-5hmC signatures enable real-time monitoring of treatment efficacy [111, 115]. This capability allows for the prediction of therapy response and assists clinicians in adjusting treatment regimens dynamically during the treatment cycle. These attributes position 5hmC as a powerful tool for addressing the challenges of tumor heterogeneity and individualizing cancer management.

Taken together, cfDNA-5hmC signatures hold significant potential as non-invasive biomarkers for tumor screening, diagnosis, monitoring, and prognosis assessment. However, its successful translation into routine clinical practice will require comprehensive validation to address key factors such as sensitivity, specificity, accessibility, patient compliance, and cost-effectiveness. Collaborative efforts to conduct large-scale, multicenter studies and establish standardized protocols will be critical in ensuring the clinical adoption of 5hmC biomarkers in cfDNA, ultimately advancing precision oncology and improving patient outcomes.

## Data Availability

No datasets were generated or analysed during the current study.

## Abbreviations

CfDNA: Cell-free DNA
5hmC: 5-Hydroxymethylcytosines
5mC: 5-Methylcytosine
cfDNA-5hmC signatures: 5hmC signatures in circulating cfDNA
TETs: Ten-eleven translocation
5fC: 5-Formylcytosine
5caC: 5-Carboxylcytosine
BS-seq: Bisulfite sequencing
C: Cytosine
T: Thymine
TAB-seq: TET-assisted bisulfite sequencing
OxBS: Oxidative bisulfite sequencing
βGT: β-Glucosyltransferase
KRuO₄: Potassium perruthenate
SMRT: Single Molecule Real-Time
LDCT: Low-dose computed tomography
AUC: Area under curve
CRC: Colorectal cancer
gFOBT: Fecal occult blood testing
FIT: Fecal immunochemical testing
MT-sDNA: Multitargeted stool DNA
CEA: Carcinoembryonic antigen
EC: Esophageal cancer
ESCC: Esophageal squamous cell carcinoma
WGS: Whole-genome sequencing
CA19-9: Carbohydrate antigen 19–9
PDAC: Pancreatic ductal adenocarcinoma
IPMNs: Intraductal papillary mucinous neoplasms
HCC: Hepatocellular carcinoma
EASL: European Association for the Study of the Liver
AASLD: American Association for the Study of Liver Diseases
AFP: Alpha-fetoprotein
DCP: Des-gamma-carboxy prothrombin
CNV: Copy number variation
IDH: Isocitrate dehydrogenase
GBM: Glioblastomas
MM: Multiple myeloma
MRD: Minimal residual disease
AML: Acute myeloid leukemia
DLBCL: Diffuse large B-cell lymphoma
FL: Follicular lymphoma
PD-L1: Programmed death-ligand 1
ICI: Immune checkpoint inhibitor
NSCLC: Non-small-cell lung cancer
LDH: Lactate dehydrogenase
LB-RECIST: Liquid Biopsy Response Evaluation Criteria in Solid Tumors
HSCT: Hematopoietic stem cell transplantation
EFS: Event-free survival
ELBS: European Liquid Biopsy Society
